# Importance of Normalization of Carbohydrate Antigen 19-9 in Patients With Intrahepatic Cholangiocarcinoma

**DOI:** 10.3389/fonc.2021.780455

**Published:** 2021-12-22

**Authors:** Hui Li, Yuan Feng, Chang Liu, Jiawang Li, Jiaxin Li, Hong Wu, Genshu Wang, Dewei Li

**Affiliations:** ^1^ Department of Liver Surgery & Liver Transplantation, West China Hospital, Sichuan University, Chengdu, China; ^2^ Department of Hepatobiliary Pancreatic Tumor Center, Chongqing University Cancer Hospital, Chongqing, China; ^3^ Department of General Surgery, Nanping District People's Hospital, Nanchong, China; ^4^ Department of Hepatic Surgery and Liver Transplantation Center, The Third Affiliated Hospital of Sun Yat-sen University, Guangzhou, China

**Keywords:** biomarker, prognosis, intrahepatic cholangiocarcinoma, carbohydrate antigen 19-9, postoperative outcomes

## Abstract

**Background:**

Although carbohydrate antigen 19-9 (CA19-9) is an established prognostic marker for intrahepatic cholangiocarcinoma (ICC) patients, the significance of elevated preoperative CA19-9 that normalized after resection remains unknown. This study aimed to investigate whether elevated preoperative CA19-9 that normalized after curative resection had an impact on prognosis among patients with ICC.

**Methods:**

Patients who underwent curative resection for stage I to III ICC between 2009 and 2018 were identified. Patients were categorized into three cohorts: normal preoperative CA19-9, elevated preoperative CA19-9 but normalized postoperative CA19-9, and persistently elevated postoperative CA19-9. Overall survival (OS), recurrence-free survival (RFS), and hazard function curves over time were analyzed.

**Results:**

A total of 511 patients (247 [48.3%] male; median age, 58 years) were included. Patients with elevated preoperative CA19-9 (n = 378) were associated with worse RFS and OS than those with normal preoperative CA19-9 (n = 152) (both *p* < 0.001). Patients with persistently elevated postoperative CA19-9 (n = 254) were correlated with lower RFS and OS than the combined cohorts with normal postoperative CA19-9 (n = 257) (both *p* < 0.001). The hazard function curves revealed that the risk of recurrence and mortality peaked earlier and higher in the elevated postoperative CA19-9 group than the other 2 groups. Multivariate analyses identified persistently elevated, rather than normalized, postoperative CA19-9 as an independent risk factor for shorter RFS and OS in ICC.

**Conclusions:**

Elevated preoperative serum CA19-9 that normalizes after curative resection is not an indicator of poor prognosis in ICC. Patients with persistently elevated postoperative CA19-9 are at increased risk of recurrence and death.

## Introduction

Intrahepatic cholangiocarcinoma (ICC) is the second most common form of primary liver cancer which accounts for 10%–15% of cases (1). The incidence of ICC has been raised in high-income countries in recent years (2). Surgical resection remains the cornerstone with a radical clearance purpose for the curative therapy of ICC. However, only approximately 20% patients are able to undergo hepatectomy and 5-year overall survival ranges from 25% to 40% after curative resection (2, 3). In addition, half of the patients experience early relapse within 2 years after surgery (4). Considering that prognoses following surgery vary considerably, surgical resection alone may be insufficient for a subset of patients (5). However, identification and selection of patients who would benefit from adjuvant therapies remain poorly defined.

Tumor markers were traditionally used for the diagnosis and estimation of prognosis for malignancies (6–8). Although there is no specific tumor marker in serum or bile, carbohydrate antigen (CA) 19-9 is the most frequently used one in the management of ICC (9, 10). A previous study has reported that elevated preoperative CA19-9 levels were associated with poor survival outcomes (11). For patients who underwent surgical resection or neoadjuvant therapies, the elevated preoperative CA19-9 could normalize after treatments (12, 13). A serum CA19-9 decline in response to neoadjuvant therapy has been associated with survival of patients with pancreatic cancer (14, 15). Yoo et al. have investigated the clinical significance of the perioperative CA19-9 change for predicting survival in 74 ICC patients and revealed that patients with normalized CA19-9 postoperatively had longer survival outcomes compared to those with persistently elevated CA19-9 (10). However, the prognostic significance of postoperatively normalized CA19-9 is still needed to be explored in a larger sample size. Furthermore, it is necessary to compare the prognosis of patients with postoperatively normalized CA19-9 or normal preoperative CA19-9.

In this study, we examined longitudinal changes in serum CA19-9 in 530 ICC patients from a multi-institutional dataset and sought to determine whether preoperative or postoperative CA19-9 was more prognostic. Specifically, we aimed to investigate whether patients with elevated preoperative CA19-9 that normalized after resection of the primary tumor were associated with similar long-term outcomes to those with normal preoperative CA19-9.

## Methods

### Study Design and Population

All consecutive patients who underwent curative resection for stage I to III ICC between January 2009 and December 2018 at Chongqing University Cancer Hospital, West China Hospital, and the Third Affiliated Hospital of Sun Yat-sen University were retrospectively reviewed. The exclusion criteria were as follows: preoperative radiofrequency ablation, transarterial chemoembolization, or other anticancer therapies; extrahepatic metastasis; patients who underwent surgical resection for tumor rupture; non-curative surgical procedures (R2); and lack of preoperative CA19-9. Patients who passed the exclusion criteria were identified and reviewed for patient demographics, clinicopathologic characteristics, perioperative clinical outcomes, and disease status at the end of follow-up. This study was approved by the Institutional Review Board of all the participating institutions, in accordance with the guidelines of the 1975 Declaration of Helsinki (16).

Preoperative CA19-9 was determined by the value closest to the time of surgical procedure, while postoperative CA19-9 was defined as the last serum CA19-9 value closest to the time of 3 months after hepatectomy and before any other adjuvant treatments. A cut-point of 22 U/ml was used to define the status of serum CA19-9 (the reference range for the CA19-9 assay was 0–22 U/ml). Patients were stratified into 3 groups by CA19-9 status as follows: patients with normal preoperative CA19-9 (normal preoperative CA19-9); patients with elevated preoperative CA19-9 but normal postoperative CA19-9 (normalized postoperative CA19-9); and patients with both elevated preoperative and postoperative CA19-9 (elevated postoperative CA19-9).

### Treatment and Follow-Up

The staging was determined by the 8th edition of the American Joint Committee on Cancer (AJCC) based on preoperative imaging including contrast-enhanced CT and magnetic resonance imaging (MRI). The lymph node examination and lymphadenectomy were performed as we previously described (17). Intraoperative ultrasound was conducted to confirm the rim of the tumor. For those with suspicious non-R0 resections, frozen sections were used to confirm the status of the surgical margin. Patients were followed by regular physical examination and serum CA19-9 per month at the first 3 months postsurgery, then every 3 to 6 months for 2 years and at 6-month intervals thereafter. Imaging, most frequently ultrasonography and contrast-enhanced CT, was performed at the time of 3 months after surgery and at least twice a year for the first 2 years. Besides, for those who determined not to go back to the hospital for reexamination, a telephone follow-up survey was performed. A definitive diagnosis of tumor relapse was based on new appearance of lesions on imaging or histological confirmation through biopsy.

### Statistical Analysis

All statistical analyses were performed by applying R (https://www.r-project.org/, version 3.3.4) and SPSS (version 23.0, SPSS Inc., Chicago, IL, USA). Continuous variables were presented as median (interquartile range [IQR]) or mean (standard deviation [SD]) and compared using the Mann–Whitney U-test. Categorical variables were compared using the chi-square or Fisher’s exact test. Overall survival (OS) and recurrence-free survival (RFS) were estimated by the Kaplan–Meier method and tested by the log-rank test. The hazard function of recurrence or death was plotted using the kernel-smoothing method (18, 19). Proportional hazard regression analyses were used for evaluation of prognostic factors of OS and RFS. Those variables with a *p* value less than 0.2 in the univariate analyses were subjected to the multivariate model. A two-sided *p* value less than 0.05 was considered statistically significant.

## Results

### Patient Characteristics

**Figure 1:**
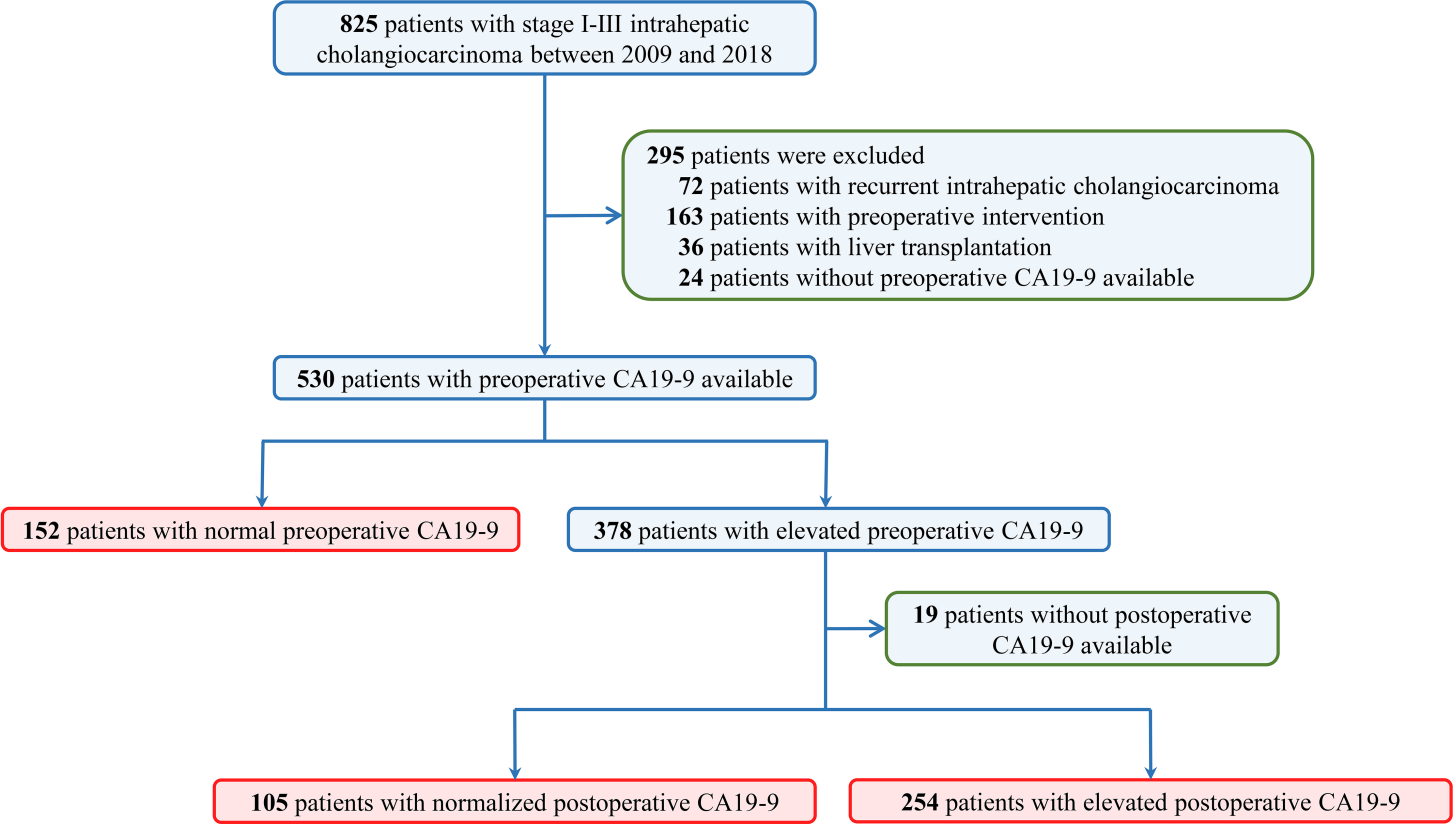
Flow diagram of the study design. Datasets were queried for patients who underwent curative hepatectomy for stage I to III intrahepatic cholangiocarcinoma from January 2009 through December 2018 in three tertiary hospitals.

A total of 825 consecutive patients who underwent curative resection for stage I to III intrahepatic cholangiocarcinoma were reviewed. A total of 295 patients were excluded due to recurrent ICC (n = 72), preoperative interventions (n = 162), liver transplantation (n = 36), or lack of preoperative CA19-9 (n=24). Among the remaining 530 patients, 152 patients (28.7%) were associated with normal preoperative CA19-9 value and 378 patients (71.3%) were with elevated CA19-9. Of the 378 patients with elevated preoperative CA19-9, postoperative CA19-9 was normalized in 105 patients and elevated in 254 patients (**Figure 1**).

**Table 1** summarizes the descriptive statistics for 511 ICC patients (male 247 [48.3%]) with either normal preoperative CA19-9 or elevated preoperative CA19-9 with evaluable postoperative CA19-9 levels. The morphologic type for tumors was 441 (86.3%) in mass-forming or intraductal growth. The median (IQR) of interval from hepatectomy to postoperative CA19-9 testing was 83 (74–97) days. With a median follow-up period of 24.8 (IQR, 12.8-37.6) months, 309 (60.5%) patients died and 371 (72.6%) patients experienced tumor relapse.

**Table 1:** Characteristics of patients.

Characteristics	All Patients(n = 511)	Normal Preoperative CA19-9 (n = 152)	Normalized Postoperative CA19-9 (n = 105)	Elevated Postoperative CA19-9 (n = 254)	p value
Gender, n (%)					0.063
Male	247 (48.3)	78 (51.3)	40 (38.1)	129 (50.8)	
Female	264 (51.7)	74 (48.7)	65 (61.9)	125 (49.2)	
Age, n (%)					0.237
<60	277 (54.2)	91 (59.9)	53 (50.5)	133 (52.4)	
≥60	234 (45.8)	61 (40.1)	52 (49.5)	121 (47.6)	
HBsAg, n (%)					0.212
Positive	145 (28.4)	49 (32.2)	25 (23.8)	71 (28.1)	
Negative	366 (71.6)	103 (67.8)	80 (76.2)	183 (71.9)	
Hepatolithiasis					0.243
Present	88 (17.2)	22 (14.5)	15 (14.3)	51 (20.1)	
Absent	423 (82.8)	130 (85.5)	90 (85.7)	203 (79.9)	
Cirrhosis					0.278
Present	139 (27.2)	44 (28.9)	22 (21.0)	73 (28.7)	
Absent	372 (72.8)	108 (71.1)	83 (79.0)	181 (71.3)	
Morphologic type					
MF, IG	441 (86.3)	131 (86.2)	86 (81.9)	224 (88.2)	
PI, MF+PI	70 (13.7)	21 (13.8)	19 (18.1)	30 (11.8)	
Tumor number					0.477
Solitary	151 (29.5)	46 (30.3)	26 (24.8)	79 (31.1)	
Multiple	360 (70.5)	106 (69.7)	79 (75.2)	175 (68.9)	
Tumor size, cm					0.162
<5	219 (42.9)	74 (48.7)	39 (37.1)	106 (41.7)	
≥5	292 (57.1)	78 (51.3)	66 (62.9)	148 (58.3)	
Differentiation					0.241
Well	22 (4.3)	9 (5.9)	6 (5.7)	7 (2.8)	
Moderate/poor	489 (95.7)	143 (94.1)	99 (94.3)	247 (97.2)	
Liver capsule invasion					0.039
Present	326 (63.8)	85 (55.9)	74 (70.5)	167 (65.7)	
Absent	185 (36.2)	67 (44.1)	31 (29.5)	87 (34.3)	
Perineural invasion					0.118
Present	77 (15.1)	16 (10.5)	15 (14.3)	46 (18.1)	
Absent	434 (84.9)	136 (89.5)	90 (85.7)	208 (81.9)	
Major vascular invasion					0.006
Present	116 (22.7)	22 (14.5)	22 (21.0)	72 (28.3)	
Absent	395 (77.3)	130 (85.5)	83 (79.0)	182 (71.7)	
MVI					0.102
Present	52 (10.2)	12 (7.9)	7 (6.7)	33 (13.0)	
Absent	459 (89.8)	140 (92.1)	98 (93.3)	221 (87.0)	
T stage					0.212
T1a	70 (13.7)	29 (19.1)	12 (11.4)	29 (11.4)	
T1b	38 (7.4)	12 (7.9)	9 (8.6)	17 (6.7)	
T2	74 (14.5)	25 (16.4)	9 (8.6)	40 (15.7)	
T3	323 (63.2)	84 (55.3)	74 (70.5)	165 (65.0)	
T4	6 (1.2)	2 (1.3)	1 (1.0)	3 (1.2)	
Lymph node invasion					0.001
Present	126 (24.7)	22 (14.5)	23 (21.9)	80 (31.5)	
Absent	385 (75.3)	130 (85.5)	82 (78.1)	174 (68.5)	
AJCC 8th edition stage					0.005
IA	59 (11.5)	25 (16.4)	10 (9.5)	24 (9.4)	
IB	35 (6.8)	12 (7.9)	9 (8.6)	14 (5.5)	
II	54 (10.6)	20 (13.2)	6 (5.7)	28 (11.0)	
IIIA	235 (46.0)	72 (47.4)	56 (53.3)	107 (42.1)	
IIIB	128 (25.0)	23 (15.1)	24 (22.9)	81 (31.9)	
Days from surgery to CA19-9 testing, median (IQR)	83 (78–97)	81 (76–93)	85 (78–102)	84 (75–98)	
Adjuvant chemotherapy					0.421
Yes	122 (23.9)	31 (20.4)	18 (17.1)	73 (28.7)	
No	344 (67.3)	109 (71.7)	78 (74.3)	157 (61.8)	
Unknown	45 (8.8)	12 (7.9)	9 (8.6)	24 (9.5)	

Data were presented as number (percentage).

ICC, intrahepatic cholangiocarcinoma; MVI, microvascular invasion; CA19-9, carbohydrate antigen 19-9; MF, mass-forming; IG, intraductal growth; PI, periductal infiltrating; AJCC, American Joint Committee on Cancer; IQR, interquartile range.

### Prognostic Importance of Normalization of CA19-9

**Figure 2:**
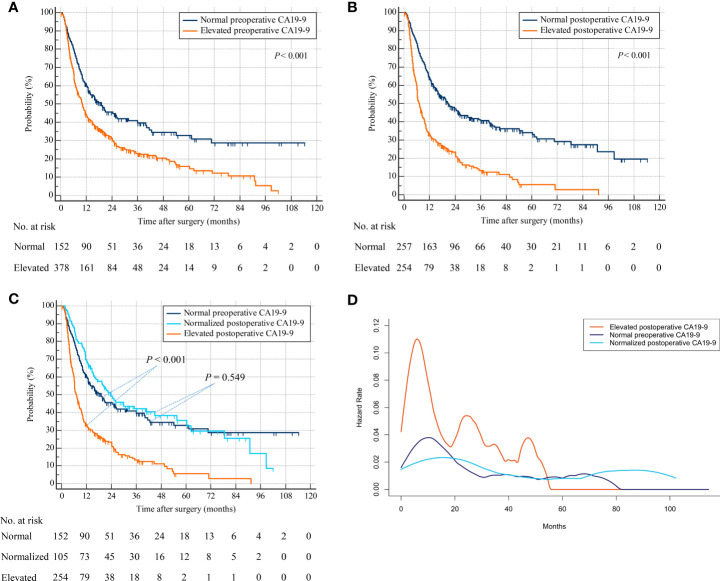
Recurrence-free survival by preoperative and postoperative CA19-9 levels. **(A)** Patients with normal versus elevated preoperative CA19-9. **(B)** Patients with normal versus elevated postoperative CA19-9. **(C)** Patients with normal preoperative versus normalized or persistently elevated postoperative CA19-9. **(D)** Hazard functions for tumor relapse in the three patient cohorts.

A total of 378 patients with elevated preoperative CA19-9 were associated with decreased RFS compared with 152 patients with normal preoperative CA19-9 (hazard ratio [HR], 1.613; 95% confidence interval [CI], 1.305–1.993; *p* < 0.001) (**Figure 2A**). The CA19-9 levels remained elevated in 254 patients after hepatectomy, while they were normalized in 105 patients with elevated preoperative CA19-9. Patients with elevated postoperative CA19-9 were correlated with worse RFS than were a total of 257 patients with normal postoperative CA19-9 (HR, 2.257; 95% CI, 1.828–2.787; *p* < 0.001) (**Figure 2B**). A total of 105 patients with normalized postoperative CA19-9 were related to statistically indistinguishable RFS from 152 patients whose CA19-9 levels remained normal after surgery (HR, 0.908; 95% CI, 0.662–1.245; *p* = 0.549) (**Figure 2C**). Importantly, 254 patients with persistently elevated CA19-9 levels after hepatectomy were associated with significantly lower RFS than those with normal preoperative or normalized postoperative CA19-9 (elevated postoperative CA19-9 vs. normal preoperative CA19-9: HR, 2.257; 95% CI, 1.760–2.895; *p* < 0.001 and elevated postoperative CA19-9 vs. normalized postoperative CA19-9: HR, 2.385; 95% CI, 1.847–3.080; *p* < 0.001) (overall log-rank *p* < 0.001) (**Figure 2C**). A repeat analysis using a CA19-9 cutoff value of 37 U/ml demonstrated similar results (**Supplementary Figure S1A**).

The smoothed hazard function curves demonstrated that the risk of ICC recurrence was peaked earlier as well as higher in the elevated postoperative CA19-9 group compared to the other 2 groups (**Figure 2D**). Furthermore, the hazard function curves for the normal preoperative and normalized postoperative CA19-9 groups were indistinguishable, suggesting a similar risk and timing of tumor relapse in these 2 groups.

**Figure 3:**
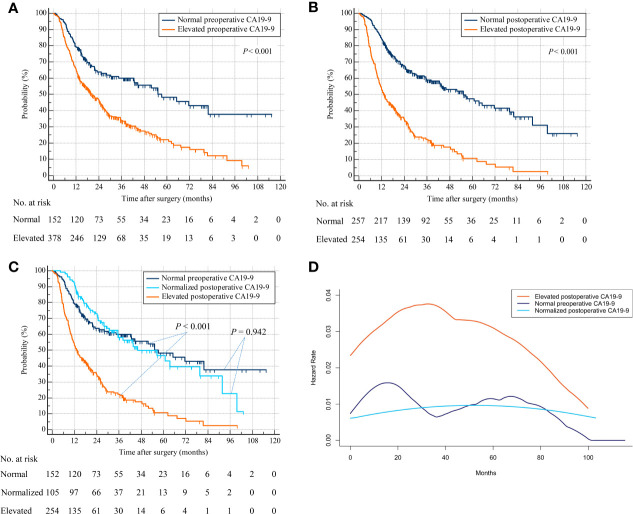
Overall survival by preoperative and postoperative CA19-9 levels. **(A)** Patients with normal versus elevated preoperative CA19-9. **(B)** Patients with normal versus elevated postoperative CA19-9. **(C)** Patients with normal preoperative versus normalized or persistently elevated postoperative CA19-9. **(D)** Hazard functions for death in the three patient cohorts.

Repeat analyses using overall survival status as endpoint produced similar results. Patients with elevated preoperative CA19-9 were associated with decreased OS compared with patients with normal preoperative CA19-9 (HR, 2.056; 95% CI, 1.632–2.589; *p* < 0.001) (**Figure 3A**). Patients with elevated postoperative CA19-9 were relevant with lower OS than patients with normal postoperative CA19-9 (HR, 2.878; 95% CI, 2.284–3.625; *p* < 0.001) (**Figure 3B**). Additionally, patients with normalized postoperative CA19-9 were related to statistically indistinguishable OS from patients whose CA19-9 levels remained normal after surgery (HR, 0.986; 95% CI, 0.681–1.428; *p* = 0.942) (**Figure 3C**). Consistently, patients with persistently elevated CA19-9 levels after hepatectomy were associated with significantly worse OS than those with normal preoperative or normalized postoperative CA19-9 (elevated postoperative CA19-9 vs. normal preoperative CA19-9: HR, 3.005; 95% CI, 2.259–3.997; *p* < 0.001 and elevated postoperative CA19-9 vs. normalized postoperative CA19-9: HR, 2.898; 95% CI, 2.186–3.843; *p* < 0.001) (overall log-rank *p* < 0.001) (**Figure 3C**). Consistently, a repeat analysis using 37 U/ml as the cutoff value of CA19-9 produced a similar result in that patients with elevated postoperative CA19-9 were associated with worse OS than those with normal preoperative CA19-9 and normalized postoperative CA19-9 (**Supplementary Figure S1B**). The smoothed hazard function curves also revealed a higher and earlier peak for mortality risk in the elevated postoperative CA19-9 group than the other 2 groups, and a similar risk and timing for death in normal preoperative and normalized postoperative CA19-9 groups (**Figure 3D**).

As shown in **Table 1**, 122 (23.9%) patients were treated with adjuvant chemotherapy. Among them, 31 had normal preoperative CA19-9, 18 were associated with normalized postoperative CA19-9, and 73 were associated with persistently elevated postoperative CA19-9. No one was treated with adjuvant radiotherapy. The Kaplan–Meier curve showed that patients treated with adjuvant chemotherapy were associated with comparable RFS compared to those without (**Supplementary Figure S2A**). Similarly, OS was comparable between patients received adjuvant chemotherapy or not (**Supplementary Figure S2B**).

Univariate analysis for RFS revealed that 11 of 15 variables were potentially predictive indicators with a *p* value less than 0.2. Subsequent multivariate analysis demonstrated that elevated postoperative CA19-9 (HR, 2.121; 95% CI, 1.650–2.727; *p* < 0.001), but not normalized postoperative CA19-9 (HR, 0.900; 95% CI, 0.654–1.239; *p* = 0.520), was an independent risk factor for poor RFS. In addition, 9 potential prognostic parameters were included in multivariate analyses for OS. Consistently, elevated postoperative CA19-9 (HR, 2.928; 95% CI, 2.193–3.909; *p* < 0.001), but not normalized postoperative CA19-9 (HR, 1.035; 95% CI, 0.712–1.504; *p* = 0.858), was an independent risk factor for poor OS in patients with ICC (**Table 2**).

**Table 2:** Prognostic factor analysis for recurrence-free survival and overall survival.

Variables	Recurrence-Free Survival				Overall Survival			
	Univariate		Multivariate		Univariate		Multivariate	
	HR (95% CI)	p value	HR (95% CI)	p value	HR (95% CI)	p value	HR (95% CI)	p value
Gender (F/M)	0.882 (0.721–1.078)	0.221			0.821 (0.659–1.024)	0.080	0.867 (0.692–1.086)	0.214
Age (≥60/<60)	0.926 (0.756–1.133)	0.454			0.956 (0.766–1.193)	0.692		
HBsAg	1.213 (0.975–1.508)	0.083	1.249 (0.997–1.563)	0.053	1.127 (0.887–1.433)	0.328		
Hepatolithiasis	0.879 (0.669–1.157)	0.359			1.200 (0.907–1.587)	0.201		
Cirrhosis	1.103 (0.882–1.378)	0.391			1.226 (0.964–1.560)	0.096	1.309 (1.021–1.678)	0.034
Tumor number (multiple/solitary)	1.873 (1.516–2.314)	<0.001	1.683 (1.352–2.094)	<0.001	1.746 (1.387–2.200)	<0.001	1.694 (1.332–2.153)	<0.001
Tumor size (≥5/<5)	1.441 (1.171–1.772)	0.001	1.248 (1.004–1.551)	0.046	1.251 (0.998–1.567)	0.052	1.156 (0.913–1.465)	0.229
Differentiation (moderate-poor/well)	4.656 (2.076–10.443)	<0.001	3.206 (1.410–7.288)	0.005	4.187 (1.729–10.139)	0.002	2.864 (1.167–7.031)	0.022
Liver capsule invasion	1.202 (0.972–1.487)	0.089	0.711 (0.488–1.037)	0.076	1.106 (0.879–1.393)	0.390		
Perineural invasion	1.268 (0.962–1.671)	0.093	1.324 ()0.986–1.778	0.062	1.541 (1.148–2.069)	0.004	1.249 (0.885–1.761)	0.206
Major vascular invasion	1.121 (0.883–1.422)	0.347			1.178 (0.912–1.522)	0.211		
MVI	1.986 (1.468–2.686)	<0.001	1.475 (1.071–2.030)	0.017	1.764 (1.273–2.444)	0.001	1.570 (1.152–2.138)	0.004
AJCC 8th edition stage	1.397 (1.111–1.757)	0.004	1.498 (0.999–2.246)	0.051	1.332 (1.038–1.710)	0.024	1.082 (0.834–1.403)	0.553
Morphologic type (PI, MF+PI/MF, IG)	1.402 (1.079–1.821)	0.011	1.012 (0.960–1.068)	0.650	1.344 (1.069–1.691)	0.012	1.172 (0.931–1.476)	0.176
CA19-9 group								
Normal preoperative	Ref				Ref			
Normalized postoperative	0.908 (0.662–1.245)	0.549	0.900 (0.654–1.239)	0.520	0.986 (0.681–1.428)	0.942	1.035 (0.712–1.504)	0.858
Elevated postoperative	2.257 (1.760–2.895)	<0.001	2.121 (1.650–2.727)	<0.001	3.005 (2.259–3.997)	<0.001	2.928 (2.193–3.909)	<0.001

ICC, intrahepatic cholangiocarcinoma; M, male; F, female; MVI, microvascular invasion; CA19-9, carbohydrate antigen 19-9; MF, mass-forming; IG, intraductal growth; PI, periductal infiltrating; Ref, reference; HR, hazard ratio; CI, confidence interval.

Analyses for metastatic recurrence sites in relation to CA19-9 status revealed that a higher frequency (83.5%) for relapse was observed in elevated postoperative CA19-9 than the other 2 groups (**Table 3**). More than half of the recurrent tumors were located only in the liver. However, no significant association was noted between normal or elevated perioperative CA19-9 and tumor recurrence sites.

**Table 3:** Analysis of sites of metastatic recurrence in relation to CA19-9 status.

Characteristics	All Patients(n = 511)	Normal Preoperative CA19-9 (n = 152)	Normalized Postoperative CA19-9 (n = 105)	Elevated Postoperative CA19-9 (n = 254)	p value
All sites	371 (72.6%)	93 (61.2%)	66 (62.9%)	212 (83.5%)	<0.001
Liver-only	231 (62.3%)	54 (58.1%)	40 (60.6%)	138 (65.1%)	0.585
Other sites					0.759
Lung	68 (18.3%)	18 (19.4%)	10 (15.2%)	40 (18.9%)	
Peritoneum	43 (11.6%)	12 (12.9%)	7 (10.6%)	24 (11.3%)	
Lymph node	42 (11.3%)	14 (15.1%)	6 (9.1%)	22 (10.4%)	
Gut	57 (15.4%)	15 (16.1%)	10 (15.2%)	32 (15.1%)	
Others	39 (10.5%)	10 (10.7%)	8 (12.1%)	21 (9.9%)	

## Discussion

Consistent with previous studies (9, 11, 20), our results showed that elevated preoperative CA19-9 correlated with poor prognosis in ICC. More importantly, postoperative CA19-9 was more informative than preoperative CA19-9. Patients with elevated preoperative CA19-9 were associated with worse OS and more frequent recurrence than those with normal preoperative CA19-9. Following curative resection, CA19-9 normalized in appropriately 30% of patients, and the survival outcomes of patients with normalized postoperative CA19-9 were similar to those with normal preoperative CA19-9. Conversely, patients with persistently elevated CA19-9 after surgery were relevant to shorter OS as well as lower RFS than those with either normal preoperative CA19-9 or normalized postoperative CA19-9. The prognostic significance of elevated postoperative CA19-9 was further revealed in a review of hazard function curves over time, which suggested a higher and earlier peak for relapse risk in the elevated operative CA19-9 group compared to the normal preoperative and normalized postoperative CA19-9 groups. Multivariate Cox regression models identified that elevated postoperative CA19-9 was more informative than elevated preoperative CA19-9 in predicting OS and tumor recurrence.

Our findings suggested that measurement of postoperative CA19-9 was more instructive than preoperative CA19-9 with regard to prognosis of ICC patients. However, this does not mean that measurement of preoperative CA19-9 can be eliminated. Preoperative CA19-9 has been associated with diagnostic utility for ICC, although both sensitivity and specificity are less than 65% (21, 22). The widespread application of high-quality computed tomography (CT) limits the utility of measuring preoperative CA19-9 in the diagnosis and evaluation of metastasis for ICC (23). Clinically, an elevated preoperative CA19-9 can be accompanied by a normal CT scan in the liver because obstruction and acute cholangitis could also result in elevation in serum CA19-9. On another aspect, a normal preoperative CA19-9 cannot eliminate a diagnosis of ICC, especially for those with a positive CT scan. In addition, guidelines do not support serological CA19-9 as a specific tumor marker for the diagnosis of ICC (21). In the present study, there were 152 patients with normal CA19-9 at the diagnosis of ICC.

Some may argue that preoperative CA19-9 could be a useful tumor marker in the follow-up of cholangiocarcinoma (9, 11). In a study of 588 patients from multi-institutional databases, by using a cutoff value of 176.3 U/ml, 146 patients with elevated preoperative CA19-9 were associated with worse prognosis compared with those with CA19-9 less than 176.3 (11). However, stratified using preoperative CA19-9 alone, patients with elevated preoperative CA19-9 were considered as a whole cohort with equally poor prognosis. In fact, an elevated preoperative CA19-9 could normalize after removal of primary tumor. Moreover, little is known regarding long-term survival as well as recurrence outcomes of these patients with normalized postoperative CA19-9 after surgery. In the present cohort, serum CA19-9 normalized in appropriately 30% of patients. Survival analyses demonstrated that patients with normalized postoperative CA19-9 were associated with comparable OS and RFS to those with normal preoperative CA19-9, suggesting that elevated preoperative CA19-9 was not informative when postoperative CA19-9 was normal.

There is no agreement whether postoperative adjuvant treatment is of positive significance for patients with ICC. The previous BILCAP study found a significantly prognostic benefit of postoperative adjuvant chemotherapy in 447 patients with biliary tract cancer (85 ICC patients) (24), while the PRODIGE 12 study demonstrated no benefit of adjuvant gemcitabine and oxaliplatin chemotherapy in 196 patients with resected biliary tract cancer (90 ICC patients) (25). In the current study, patients treated with adjuvant chemotherapy were associated with comparable OS and RFS compared to those without, implying no prognostic benefit of adjuvant chemotherapy for resected ICC in our dataset. However, our results were based on retrospective analysis which contained confounders. Future RCT studies with large-scale sample are still needed to further clarify the prognostic significance of adjuvant treatments for ICC.

The half-life of CA19-9 varies from less than 1 day to about 3 days (26). To ensure sufficient time to allow CA19-9 normalization, we limited the timing of postoperative CA19-9 measurement to 3 months after hepatectomy and before any other adjuvant treatments (IQR, 78-97 days). In addition, we also performed survival analyses by using 1 month postsurgery as the time point for postoperative test of CA19-9. As shown in **Supplementary Figure S3**, no significant difference regarding OS and RFS was observed between 77 patients with normalized postoperative and 152 patients with normal preoperative CA19-9 but showed a tendency of worse prognosis for patients with normalized postoperative CA19-9. A previous study recommended a postoperative CA19-9 level as the lowest level during follow-up after surgery (10). In the present work, we also analyzed the correlation between the lowest CA19-9 with long-term survival and did find similar results (**Supplementary Figure S4**). In this regard, screening those with elevated postoperative CA19-9 at the 3-month time-point out then subjecting to adjuvant therapies and more frequent surveillance may be an efficient strategy for improvement of patient prognosis.

However, we could not conclude from this dataset whether additional immediate imaging examination would be beneficial for those with elevated postoperative CA19-9. We did not find a significant difference regarding sites of tumor relapse among patients with a normalized or persistently elevated CA19-9 level. A hepatobiliary ultrasound accompanied by a serum CA19-9 evaluation would be beneficial in monitoring potential recurrence. Postoperative CA19-9 might inform the frequency for surveillance. Patients with persistently increased postoperative CA19-9 level were associated with a recurrence hazard function rose and peaked quicker before declining to meet those with a normal preoperative or normalized postoperative CA19-9. This may support using the postoperative CA19-9 level to stratify patients for graded surveillance.

Although current guidelines for ICC did not recommend routine adjuvant chemotherapy for cholangiocarcinoma, a recent randomized controlled trial demonstrated a significant benefit in OS from the addition of adjuvant chemotherapy using capecitabine (24). We did not conclude that postoperative CA19-9 level could be an indicator for stratification of patients that benefited from adjuvant chemotherapy. Nevertheless, our results indicated an early evaluation of postoperative CA19-9 level, and the 3-month time point was sufficient and was beneficial for the formulation of therapeutic strategies for patients in high risk of recurrence.

This study was limited by its retrospective nature. For instance, the postoperative CA19-9 value was not available in 19 patients with elevated preoperative CA19-9. According to the follow-up strategy, CA19-9 should be evaluated at 3 months post-hepatectomy, but the timing of postoperative CA19-9 measurement was not under control. Fortunately, the postoperative CA19-9 level was routinely measured before adjuvant therapies for all the patients. Furthermore, although a large sample size from multiple centers was used for analysis, only patients in Eastern Asia were included. We did not assess the optimal cutoff value for CA19-9 but did find consistent results using the two most used cutoff values. Confirmatory studies would be necessary.

In conclusion, this study suggests that the prognostic significance of postoperative CA19-9 exceeds that of preoperative CA19-9. Patients with elevated preoperative CA19-9 that normalizes after curative resection are associated with comparable long-term survival outcomes compared to those with normal preoperative CA19-9, whereas patients with elevated postoperative CA19-9 are at increased risk of recurrence and death. Measurement of postoperative CA19-9 level may inform an individualized and risk-adjusted surveillance strategy.

## Data Availability Statement

The raw data supporting the conclusions of this article will be made available by the authors, without undue reservation.

## Ethics Statement

The studies involving human participants were reviewed and approved by the Institutional Review Board of Chongqing University Cancer Hospital. The patients/participants provided their written informed consent to participate in this study.

## Author Contributions

Conceptualization, HL, DL, GW, and HW. Data curation, HL, YF, and CL. Formal analysis, HL, YF, JxL, and JwL. Supervision, DL, GW, and HW. Writing—original draft, HL, YF, and CL. All authors contributed to the article and approved the submitted version.

## Funding

This work was supported by the National Natural Science Foundation of China (81972747, 81672882, and 81870447). Sichuan Science and Technology Plan Project (2021YFH0095).

## Conflict of Interest

The authors declare that the research was conducted in the absence of any commercial or financial relationships that could be construed as a potential conflict of interest.

## Publisher’s Note

All claims expressed in this article are solely those of the authors and do not necessarily represent those of their affiliated organizations, or those of the publisher, the editors and the reviewers. Any product that may be evaluated in this article, or claim that may be made by its manufacturer, is not guaranteed or endorsed by the publisher.

## Supplementary Material

Supplementary Figure S1 Long-term survival by preoperative and postoperative CA19-9 levels using cut-off of 37 U/ml. **(A)**, recurrence-free survival of patients with normal preoperative versus normalized or persistently elevated postoperative CA19-9. **(B)**, overall survival of patients with normal preoperative versus normalized or persistently elevated postoperative CA19-96.

Supplementary Figure S2 Kaplan-Meier curves showing long-term survival of patients treated with adjuvant chemotherapy or not. **(A)**, recurrence-free survival. **(B)**, overall survival.

Supplementary Figure S3 Long-term survival by preoperative and postoperative CA19-9 levels using one-month as time point for postoperative test of CA19-9. **(A)**, recurrence-free survival of patients with normal preoperative versus normalized or persistently elevated postoperative CA19-9. **(B)**, overall survival of patients with normal preoperative versus normalized or persistently elevated postoperative CA19-9.

Supplementary Figure S4 Long-term survival by preoperative and postoperative CA19-9 levels using lowest value during follow-up. **(A)**, recurrence-free survival of patients with normal preoperative versus normalized or persistently elevated postoperative CA19-9. **(B)**, overall survival of patients with normal preoperative versus normalized or persistently elevated postoperative CA19-9.
