# Supplementary figures and images for: Importance of Normalization of Carbohydrate Antigen 19-9 in Patients With Intrahepatic Cholangiocarcinoma
Source: Front Oncol. 2021 Dec 22;11:780455. doi: 10.3389/fonc.2021.780455 (PMC8728073; doi:10.3389/fonc.2021.780455)

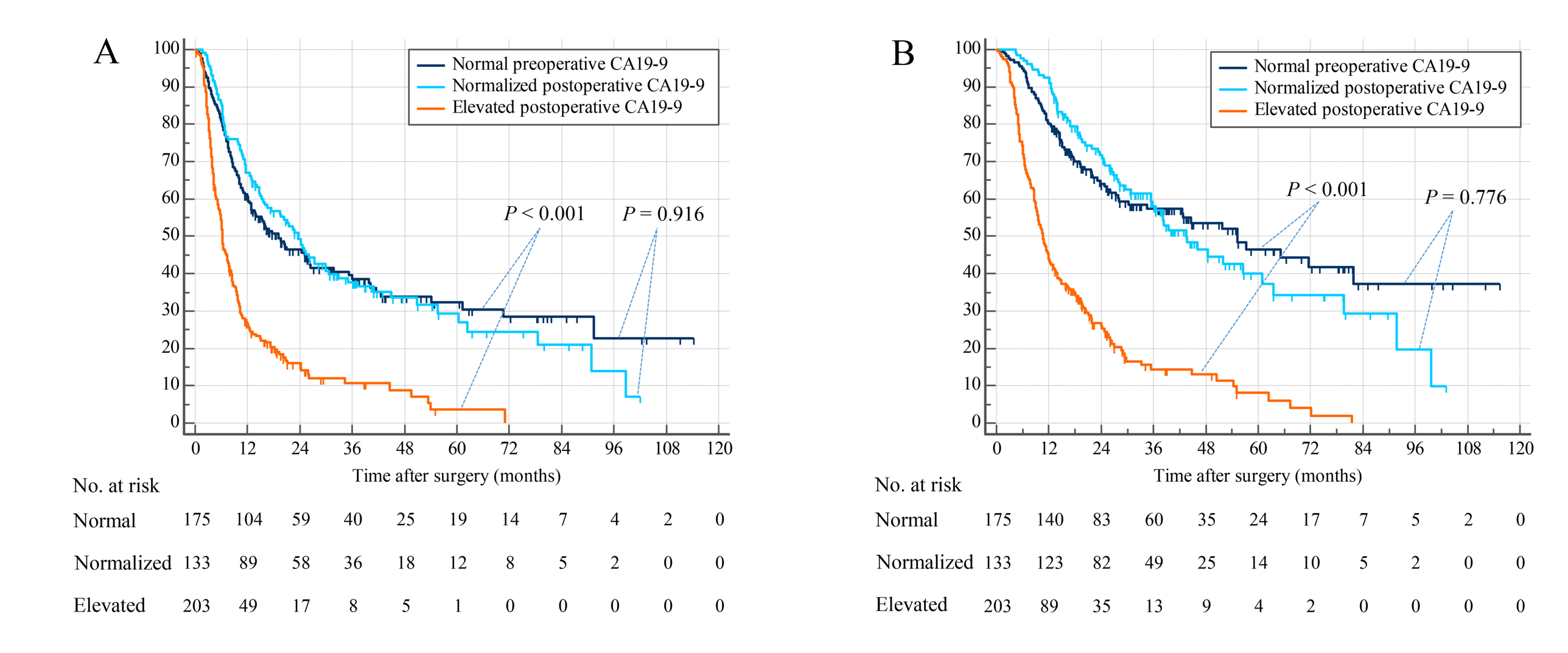

Supplement: Supplementary Figure S1 — Long-term survival by preoperative and postoperative CA19-9 levels using cut-off of 37 U/ml. (A), recurrence-free survival of patients with normal preoperative versus normalized or persistently elevated postoperative CA19-9. (B), overall survival of patients with normal preoperative versus normalized or persistently elevated postoperative CA19-96. [file Image_1.jpeg]

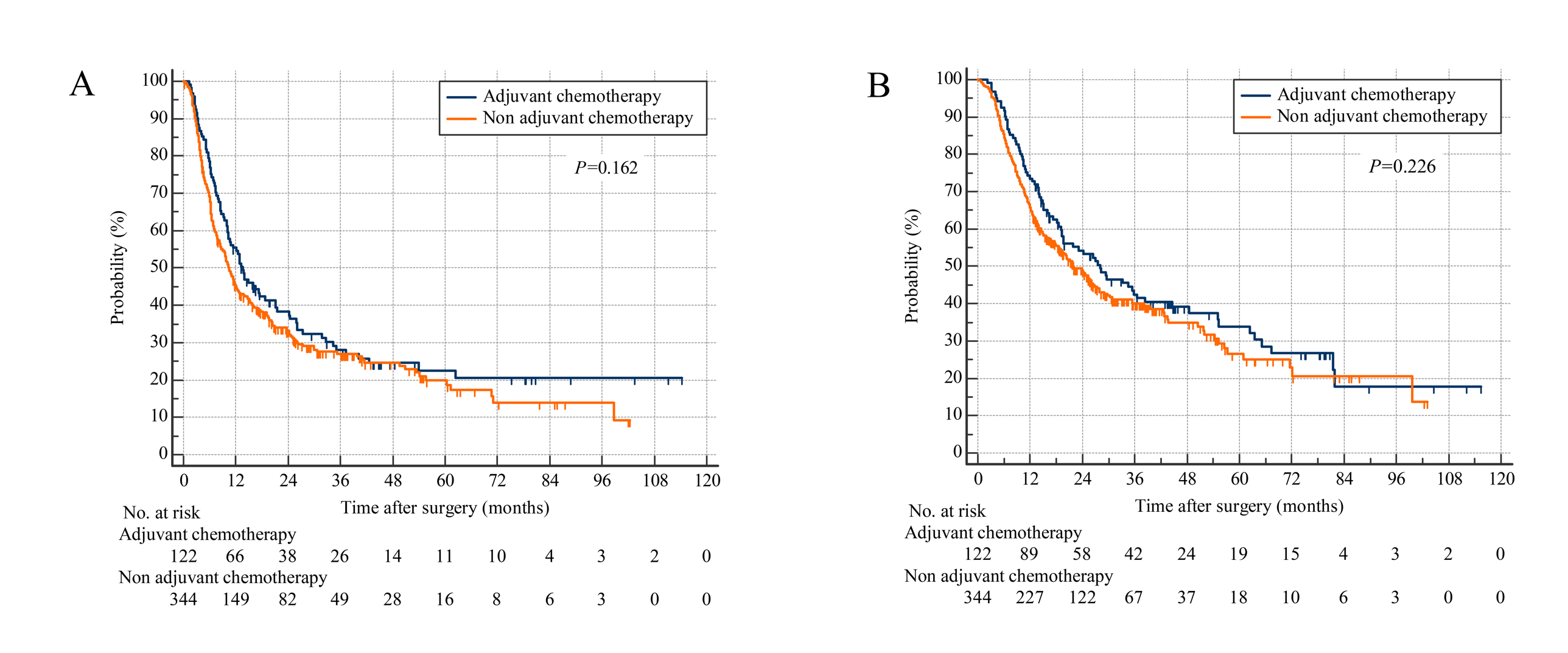

Supplement: Supplementary Figure S2 — Kaplan-Meier curves showing long-term survival of patients treated with adjuvant chemotherapy or not. (A), recurrence-free survival. (B), overall survival. [file Image_2.jpeg]

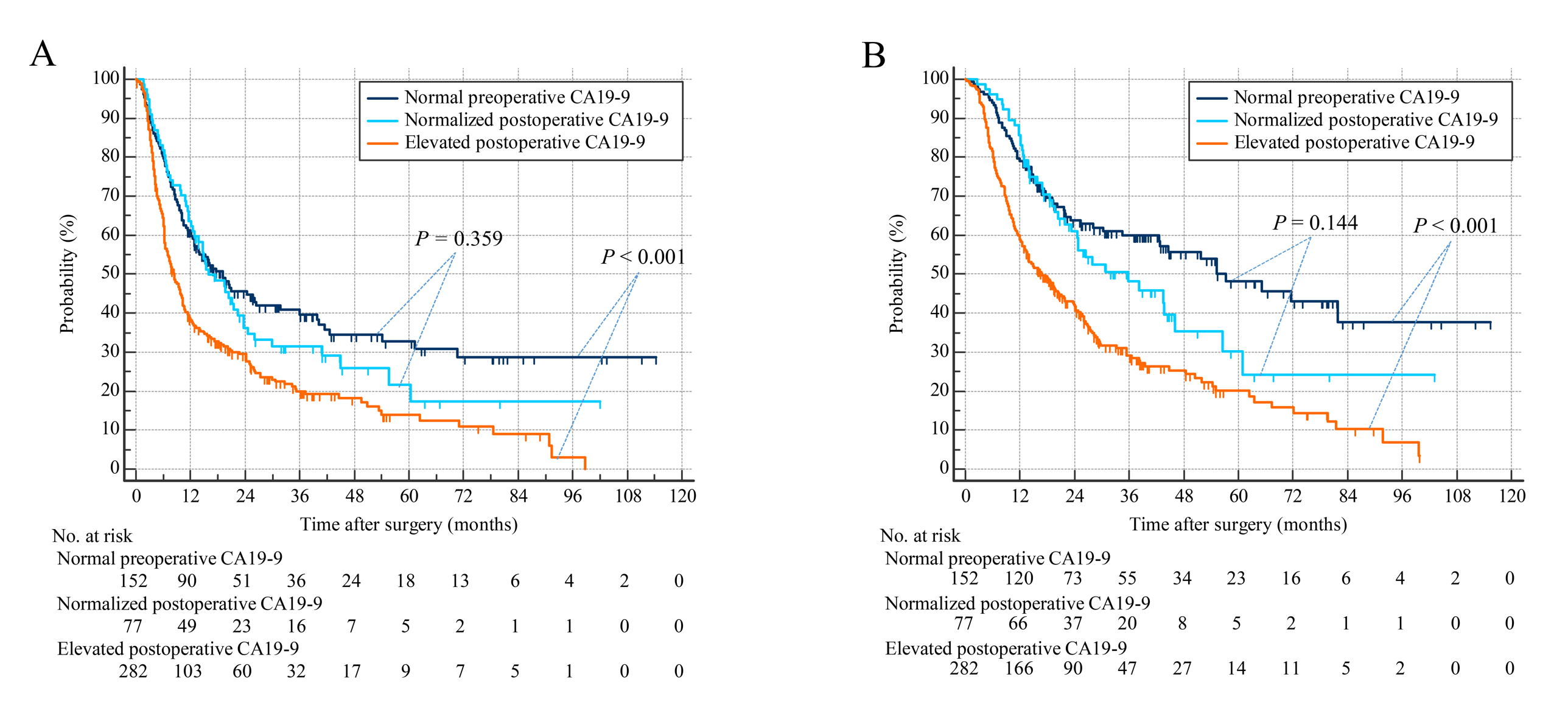

Supplement: Supplementary Figure S3 — Long-term survival by preoperative and postoperative CA19-9 levels using one-month as time point for postoperative test of CA19-9. (A), recurrence-free survival of patients with normal preoperative versus normalized or persistently elevated postoperative CA19-9. (B), overall survival of patients with normal preoperative versus normalized or persistently elevated postoperative CA19-9. [file Image_3.jpeg]

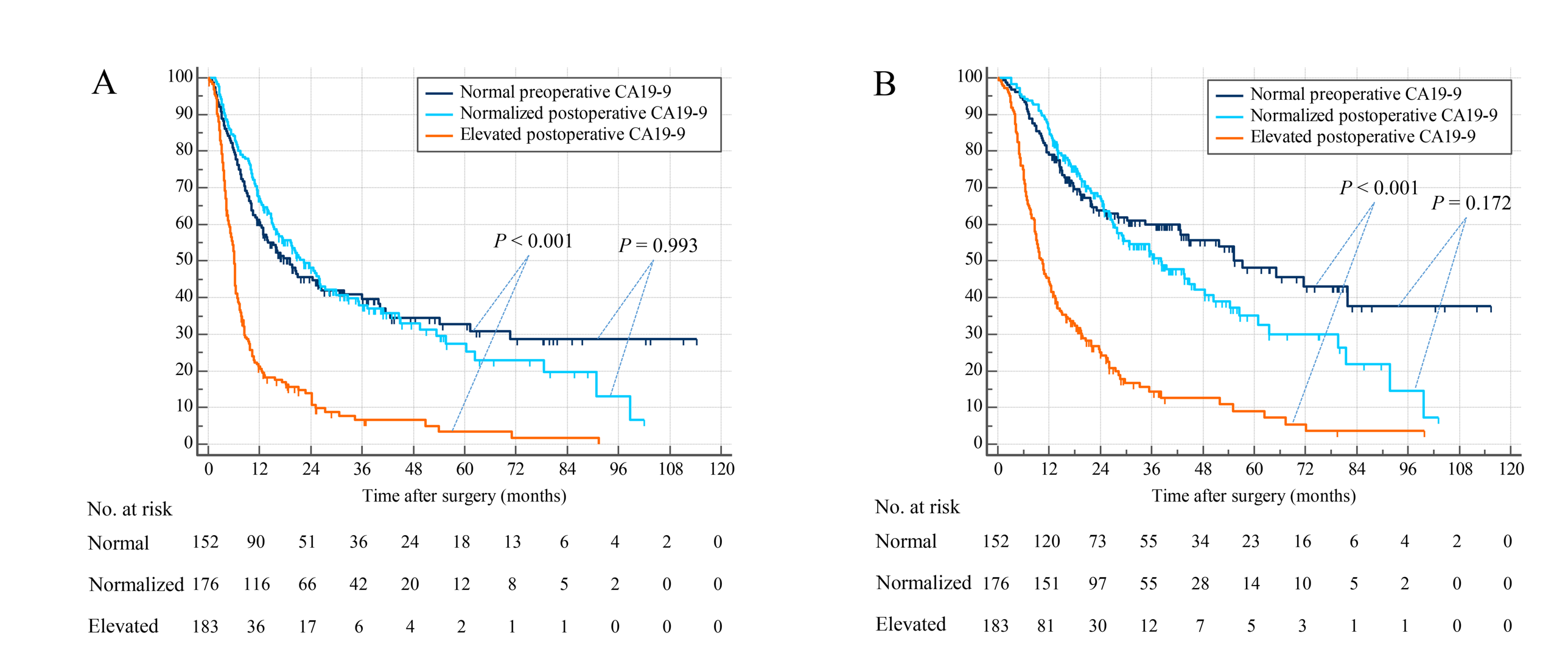

Supplement: Supplementary Figure S4 — Long-term survival by preoperative and postoperative CA19-9 levels using lowest value during follow-up. (A), recurrence-free survival of patients with normal preoperative versus normalized or persistently elevated postoperative CA19-9. (B), overall survival of patients with normal preoperative versus normalized or persistently elevated postoperative CA19-9. [file Image_4.jpeg]
